# Patient-Specific 3-Dimensional Model of Smooth Muscle Cell and Extracellular Matrix Dysfunction for the Study of Aortic Aneurysms

**DOI:** 10.1177/15266028211009272

**Published:** 2021-04-26

**Authors:** Natalija Bogunovic, Jorn P. Meekel, Jisca Majolée, Marije Hekhuis, Jakob Pyszkowski, Stefan Jockenhövel, Magnus Kruse, Elise Riesebos, Dimitra Micha, Jan D. Blankensteijn, Peter L. Hordijk, Samaneh Ghazanfari, Kak K. Yeung

**Affiliations:** 1 Amsterdam Cardiovascular Sciences, Department of Vascular Surgery, Amsterdam University Medical Centers, Location VUmc, Amsterdam, The Netherlands; 2 Amsterdam Cardiovascular Sciences, Department of Physiology, Amsterdam University Medical Centers, Location VUmc, Amsterdam, The Netherlands; 3 Amsterdam Cardiovascular Sciences, Department of Clinical Genetics, Amsterdam University Medical Centers, Location VUmc, Amsterdam, The Netherlands; 4 Optics11 BV, Amsterdam, The Netherlands; 5 Aachen-Maastricht Institute for Biobased Materials, Faculty of Science and Engineering, Maastricht University, Geleen, The Netherlands; 6 Department of Biohybrid & Medical Textiles (Biotex), RWTH Aachen University, Aachen, Germany; 7 Institut für Textiltechnik der RWTH Aachen University, Aachen, Germany

**Keywords:** aortic aneurysm, smooth muscle cell, extracellular matrix, translational research

## Abstract

**Introduction:**

Abdominal aortic aneurysms (AAAs) are associated with overall high mortality in case of rupture. Since the pathophysiology is unclear, no adequate pharmacological therapy exists. Smooth muscle cells (SMCs) dysfunction and extracellular matrix (ECM) degradation have been proposed as underlying causes. We investigated SMC spatial organization and SMC-ECM interactions in our novel 3-dimensional (3D) vascular model. We validated our model for future use by comparing it to existing 2-dimensional (2D) cell culture. Our model can be used for translational studies of SMC and their role in AAA pathophysiology.

**Materials and Methods:**

SMC isolated from the medial layer of were the aortic wall of controls and AAA patients seeded on electrospun poly-lactide-*co*-glycolide scaffolds and cultured for 5 weeks, after which endothelial cells (EC) are added. Cell morphology, orientation, mechanical properties and ECM production were quantified for validation and comparison between controls and patients.

**Results:**

We show that cultured SMC proliferate into multiple layers after 5 weeks in culture and produce ECM proteins, mimicking their behavior in the medial aortic layer. EC attach to multilayered SMC, mimicking layer interactions. The novel SMC model exhibits viscoelastic properties comparable to biological vessels; cytoskeletal organization increases during the 5 weeks in culture; increased cytoskeletal alignment and decreased ECM production indicate different organization of AAA patients’ cells compared with control.

**Conclusion:**

We present a valuable preclinical model of AAA constructed with patient specific cells with applications in both translational research and therapeutic developments. We observed SMC spatial reorganization in a time course of 5 weeks in our robust, patient-specific model of SMC–EC organization and ECM production.

## Introduction

Abdominal aortic aneurysms (AAAs) are pathological dilations of the aorta. Ruptured AAAs are associated with a mortality rate of up to 90%.^1^ The pathophysiology of AAA remains elusive.^2^ Defects in medial smooth muscle cells (SMCs),^3–6^ extracellular matrix (ECM) remodeling,^7–9^ and decreased ECM production by SMC^7,8^ are associated with AAA development. Since the pathophysiology is unclear, no pharmacological therapy is available, leaving surgery as the only treatment option.

Current guidelines for aneurysm surgery are still mostly diameter based.^10^ Research and therapy development are hindered by the difficulty of understanding the pathophysiology of the disease and lack of adequate preclinical models. Recreating the complex micro-environment of the aorta and constructing a relevant disease model could improve these prospects and therapeutic options for the patients. As cultures on plastic or glass can be maintained for a shorter period of time, due to cell detachment in overgrown layers, patterned scaffolds can be employed as an advanced alternative.^11–13^ However, bioengineered grafts to study large vessel diseases such as AAA are not available.

We aim to build a complex preclinical 3-dimesional (3D) cell culture model seeded with patient-specific SMC to study AAA pathophysiology. We aim to validate this 3D model of SMC and the ECM they produce by comparing it with existing 2D models to demonstrate increased robustness and complexity. Mechanical properties, fiber orientation, and ECM secretion were assessed to characterize the scaffolds. Scaffolds seeded with healthy SMC were compared to scaffolds seeded with SMC of AAA patients to potentially uncover AAA-related molecular defects.

## Materials and Methods

### Patient Population

AAA biopsies were collected during open aneurysm repair at the Amsterdam University Medical Centers, location VU Medical Center (Amsterdam, the Netherlands). Control biopsies were collected from nondilated aortas of heart-beating donors during organ harvesting procedures for renal transplantation. All AAA patients signed an informed consent to participate in the study. Material from control biopsies was collected anonymously with only age and gender available. All material was collected in accordance with regulations of the World Medical Association Declaration of Helsinki and institutional guidelines of the Medical Ethical Committee of the Amsterdam UMC, location VU Medical Center. Patient and control clinical information is shown in Supplementary Table 1.

### Aortic Biopsy Explant Protocol

Primary aortic SMC were isolated from aortic biopsies of healthy controls and AAA patients according to our previously described protocol.^14^ SMC of controls (n=7) and AAA patients (n=8) were used in the experiments. All SMC cell lines were cultured in 231 medium (Thermo Fisher Scientific, Waltham, MA, USA), supplemented with Smooth Muscle Growth Supplement (Thermo Fisher Scientific), and 100 units/mL penicillin and 100 µg/mL streptomycin (Thermo Fischer Scientific) to provide optimal vascular SMC growth. Cells were cultured in a humidified incubator at 37 °C, 5% CO_2_.

### Fabrication of Scaffolds

The membranes were prepared by electrospinning process, using a Fluidnatek LE500 (Bioinicia SL, Valencia, Spain provided by IuL Biosystems, Königwinter, Germany). Fibers made of poly-lactide-*co*-glycolide (PLGA; Purasorb PLG 8523, Corbion, Gorinchem, Netherlands), a biocompatible and biodegradable polymer,^15^ were spun from a solution with a mass concentration of 5 wt% PLGA in 75% chloroform (CHCl_3_, Carl Roth GmbH, Karlsruhe, Germany) and 25% methanol (MeOH, neoLab Migge Laborbedarf–Vertriebs GmbH, Heidelberg, Germany). All chemicals were used as delivered, without further purification.^16^

PLGA was dried in a vacuum at 40 °C for 12 hours before dissolution under continuous stirring for 12 hours in the mentioned solvents. In preparation of the spinning process, the spinning solution was filled into a 5-mL syringe (B. Braun Melsungen AG, Melsungen, Germany), which was connected to a multi nozzle spinneret with 24 nozzles. The polymer solution was extruded using the syringe pump at a flow rate of 25 mL/h through a hollow needle with a diameter of 0.4 mm (B. Braun Melsungen AG, Melsungen, Germany). At a voltage of ±25 kV, the polymer solution is stretched into fibers. The fibers were collected on a rotating mandrel (Ø=200 mm) at a rotational speed of 100 rpm. The nonwoven thickness was 250 to 300 µm. The spinning process was carried out at 23 °C and at 30% relative humidity. The morphology of the scaffold was examined using scanning electron microscopy (SEM; Leo 1450 VP, Zeiss, Germany; Supplementary Figure 1a).

The pore size distribution of three different samples was measured with a capillary flow pore size meter (PSM 165, Topas GmbH, Dresden, Germany). An adapter with a flow cross-section of 2.01 cm^2^ and a test fluid (Topor, Topas GmbH, Dresden, Germany) with a surface tension of 16.0 mN/m were used.^16^

### Seeding SMCs and Endothelial Cells (EC) in 2D and 3D Cultures on Scaffolds

**Figure 1.**
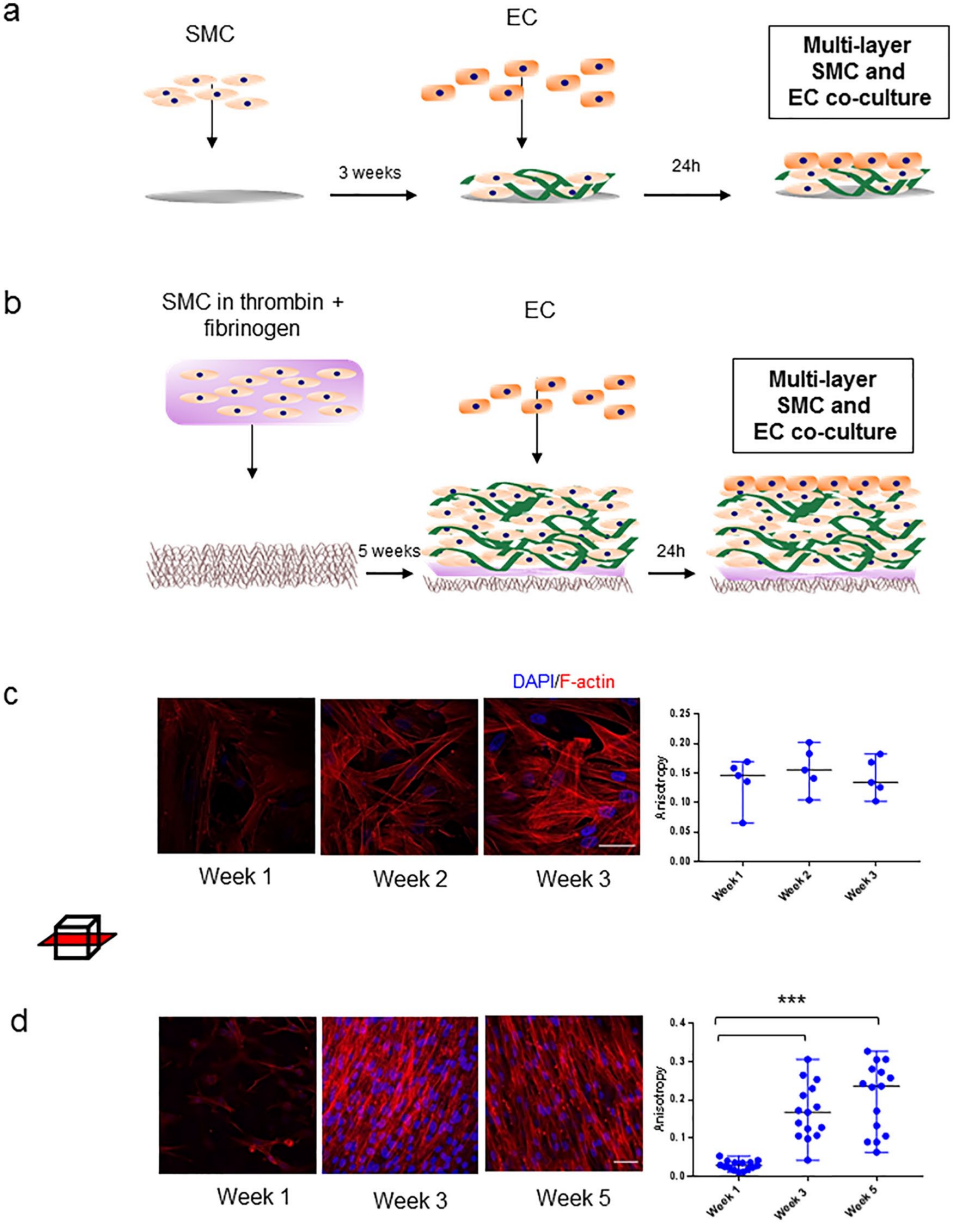
Two- and 3-dimensional smooth muscle cell (SMC) cultures. (a) Schematic of the 2D co-culture experiment with SMC and EC seeded on glass coverslips during a 3-week time course. (b) Schematic of the 3D co-culture experiment with SMC and endothelial cells (ECs) seeded on poly-lactide-*co*-glycolide (PLGA) scaffolds during a 5-week time course. Samples were immunostained for F-actin (red) and DAPI (blue). (c) Images left to right: representative images of a control SMC F-actin alignment on glass coverslips in week 1, 2, and 3. Boxplot shows anisotropy measurements of SMC F-actin alignment in time. Data points represent individual measurements in one representative control cell line per time point. (d) Images left to right: representative images of a control SMC F-actin alignment on 3D scaffold at week 1, 3, and 5. Boxplot shows anisotropy measurements of SMC F-actin alignment in time. Data points represent individual measurements in one representative control cell line per time point (***p<0.001 vs week 3; vs week 5). Graphs are shown as median with range. Scale bar: 50µm.

SMC of controls and AAA patients were seeded on round glass coverslips in 6-well plates (13 mm, #1; Thermo Fischer Scientific) in a seeding density of 100,000 cells/well in complete SMC medium, as depicted in Figure 1a.

The PLGA scaffold sheet was ultraviolet light sterilized on each side for 30 minutes and cut into ribbons of 30×5 mm in the direction parallel to the scaffold patterning, which were glued (Secondelijm, Pattex, Henkel, Amsterdam, the Netherlands) onto nylon rings (outer diameter: 30 mm, inner diameter: 19 mm, Praxis, Diemen, the Netherlands). Scaffolds were dried at room temperature for 24 hours, ultraviolet sterilized again and incubated in complete medium overnight.

The next day, complete medium was aspirated. Powder thrombin (Sigma-Aldrich) was diluted into a final concentration of 10 IU/mL, and powder fibrinogen (Sigma), to a final concentration of 10 mg/mL medium, both filter sterilized and in nonsupplemented medium kept on ice prior to seeding. Per scaffold, 450,000 cells were resuspended in 20 µL of ice-cold thrombin solution and mixed with 20 µL fibrinogen. Cells were onto the scaffold in the inner ring centralized seeding area (19×5 mm; Figure 1b, Supplementary Figure 1b). As the mixture rigidified on the scaffold, they were placed in an incubator for 30 minutes, after which complete medium was added.

After 3 or 5 weeks of culturing, ECs [human umbilical vein endothelial cells (HUVEC); Lonza, Basel, Switzerland] were added on top of the multilayer of SMC and ECM (Figure 1a and b) in 2D and 3D cultures, respectively. Two days prior to EC seeding, complete 231 SMC culture medium was exchanged for endothelial cell medium (Sanbio, Uden, the Netherlands). 300,000 ECs were seeded per well on top of the SMC. Medium was aspirated from the wells and EC were pipetted on top of the seeding surface. Two days after seeding, samples were fixated in 4% paraformaldehyde.

### Immunostaining and Confocal Microscopy

After fixation in 4% paraformaldehyde, samples were washed 3 times with phosphate buffered saline–Tween (PBST) and the cells were subsequently permeabilized for 10 minutes in 0.2% Triton in PBST. Samples were incubated in blocking solution [1% bovine serum albumin (BSA) solution in PBST for 1 hour at room temperature]. Primary rabbit monoclonal antibody against VE-cadherin (D87F2, Cell Signaling, Danvers, MA, USA) and primary mouse monoclonal antibody against fibrilin-1 (MAB2502, Merck, Kenilworth, NJ, USA) were incubated overnight at 4 °C in 1:400 dilution. Samples were washed 5 times in PBST. Secondary antibodies Alexa Fluor 488 Goat Anti-Mouse (IgG) and Alexa Fluor 546 Goat Anti-Rabbit (IgG) (1:100; Thermo Fisher Scientific), DAPI to label cell nuclei (1:200; Thermo Fisher Scientific), and Alexa Fluor 647 Phalloidin to label F-actin (1:200; Thermo Fisher Scientific), were incubated for 1 hour at room temperature in the dark. Scaffold samples were sealed with Mowiol mounting medium containing diazabicyclo-octane (Dabco, Sigma-Aldrich) in glass bottom 35-mm dishes. Coverslips were sealed with Mowiol on glass slides. Images were acquired using the Nikon A1R (Nikon, Tokyo, Japan) microscope and the corresponding Nis-Elements C Software (Nikon). Representative images were analyzed and separate channel images were created using Fiji (v.152, National Institutes of Health, Bethesda, MD, USA).^17^

### SMC F-Actin Alignment in Multilayers

Organization of the F-actin fibers was characterized by anisotropy measurements using FibrilTool plug-in in Fiji,^18^ to determine SMC organization and cell culture complexity. Values of the anisotropy index range from 0 to 1, where parallel lines result in an anisotropy index 1, and unorganized lines result in values closer to 0. F-actin alignment was measured in acquired images of SMC controls in 2D and 3D cultures. Following the published protocol, X-Y fields of view were marked as regions of interest (ROI). Using FibrilTool, anisotropy was quantified within these ROI. For 2D cultures, anisotropy was measured on single focal plane images. For 3D cultures seeded on scaffolds, anisotropy was measured on 15 optical slices throughout the Z stack with a step size 0.5 µm in 2 control and 2 patient cell lines.

### Measuring Scaffold Stiffness Using Nanoindentation

Micromechanical properties of scaffolds without cells and scaffolds seeded with control SMC were measured after 5 weeks. Scaffolds in complete cell culture medium were measured with the Piuma Nanoindenter instrument (Optics11, Amsterdam, the Netherlands). The surface of the scaffold was indented with a spherical indentation probe with a spring constant and tip radius of approximately 0.04 N/m and 61 μm, respectively. The indentation probe is part of a unique optomechanical ferrule-top cantilever force transducer, operated by a *z*-axis piezoelectric motor.^19^ A controlled piezo displacement rate of 5 µm/s was set to move the glass tip into the sample surface. The resulting force was measured through cantilever displacement. The elastic properties of the scaffolds were given by the effective (or reduced, plain strain) Young’s modulus, which does not include any assumption for the Poisson ratio of the scaffolds. The effective Young’s modulus was modeled within the linear elastic regime, up to an indentation depth of 1 μm for the scaffolds without cells, and 2 µm for the cell-containing scaffolds. For both samples, 25 measurements were taken from a grid scan with an area of 400 µm × 400 µm. Hertz contact model was applied to the loading curve, as suggested for measuring viscoelastic materials, and denotes the effective Young’s modulus (*E*_eff_) as shown in Equation 1, with *R* as indenter tip radius, *h* as indentation depth and *P* as load.^20,21^

(1) $$E_{\mathrm{eff}}=\frac{3}{\sqrt{4PR}\times \sqrt[{3/2}]{h}}$$

### Fibrilin-1 Production by SMC

Fibrilin-1 production by SMC was semiquantitatively assessed using the acquired immunostaining images. The area of fibrilin-1 fibers in each field of view ROI was masked by adjusting the threshold for the 488 fluorescent channel. Acquired area values in the ROI were normalized by dividing the area value by the number of nuclei per image. Fibrilin-1 production was compared between 2 control and 2 patient cell lines in 3D cultures after 5 weeks.

### Mechanical Properties and Tensile Strength

Tensile stress and elastic modulus were compared between control (n=5) and patient (n=5) cell lines in 3D scaffolds after 5 weeks of cultures. Means of 3 individual scaffolds per cell line were used to calculate the final data points. Uniaxial tensile tests on the samples were performed using a mechanical tester (Zwick, Ulm, Germany) at a strain rate of 3 mm/min with a 10 N load cell. The dimensions of the samples were measured using a digital caliper before each test. Ultimate tensile stress and the elastic modulus (*E*) were calculated from the stress-strain curve of each measurement. The elastic modulus was determined via linear regression of the slope of the curve.

### Statistical Analysis

Data were analyzed with SPSS (v22.0, IBM, Armonk, NY, USA). Two groups were compared using the nonparametric Mann-Whitney *U* test and multiple groups were compared using Kruskal-Wallis test and using the Mann-Whitney *U* test for post hoc analysis. Correlations were tested with Spearman’s rank correlations. Multiple related samples were tested with Friedman’s analysis of variance. Data are presented as box plots with median and range. Tests were considered statistically significant at p<0.05. Boxplots and scatterplots were made using GraphPad Prism7 (GraphPad Software Inc, San Diego, CA, USA). If the number of samples was low, individual data was presented.

## Results

### SMC Organization and F-Actin Alignment in 2D and 3D cultures

SMC seeded in 2D cultures adhered throughout the surface of the glass coverslip after 1 week, and continued proliferating into multiple layers throughout weeks 2 and 3. F-actin alignment over time was quantified. SMC show random F-actin alignment on the coverslip after 1 week. F-actin parallel organization did not increase in weeks 2 and 3 compared to week 1 (Figure 1c). SMC seeded on 3D scaffolds developed confluent monolayers on top of each other, mimicking the aortic media. F-actin parallel organization progressively increased in time: Week 1 showed a mean anisotropy of 0.02 vs week 3 0.17 (p<0.001) vs week 5 0.20 (p<0.001; Figure 1d). After week 3, the anisotropy did not change further.

### Endothelial Monolayer Morphology in 2D and 3D Cultures

**Figure 2.**
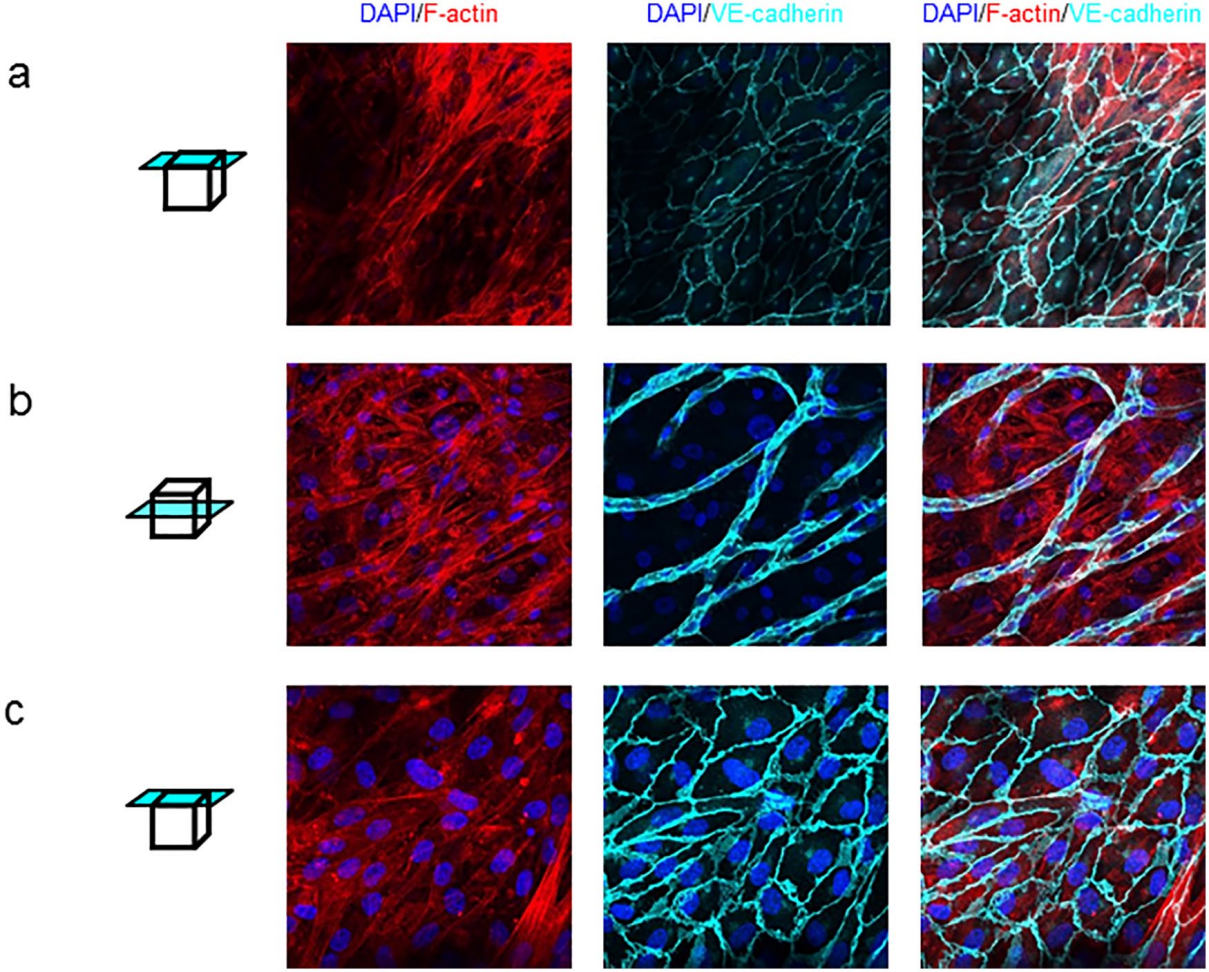
Endothelial cell (EC) monolayer morphology in 2-dimensional (2D) and 3-dimensional (3D) smooth muscle cell (SMC) cultures. (a) Representative images of a co-culture of multilayered SMC and a monolayer of EC on top of them, seeded on a glass coverslip (top layer). (b) Representative images of a co-culture of multilayered SMC and EC forming tube-like structures on a 3D scaffold (middle layer). (c) Representative images of a co-culture of multilayered SMC and a monolayer of EC on top of them, seeded on a 3D scaffold (top layer). Representative images of merge F-actin and DAPI, VE-cadherin and DAPI and merge tricolor. Scale bar: 50 µm.

ECs were seeded on top of the multilayered SMC in 2D and 3D cultures after 3 and 5 weeks, respectively. EC formed a confluent monolayer on the SMC 2D culture, characterized by VE-cadherin staining (Figure 2a). ECs seeded on 3D scaffolds, and thus more layers of SMC, exhibit diverse morphologies in different regions. In some regions, EC grew on top of the SMC multilayers, forming tube-like structures, partially overgrown SMC (Figure 2b). Other regions on the scaffolds were distinguishable by a confluent monolayer of EC representing the endothelial barrier (Figure 2c).

### Stiffness and Viscoelastic Behavior

**Figure 3.**
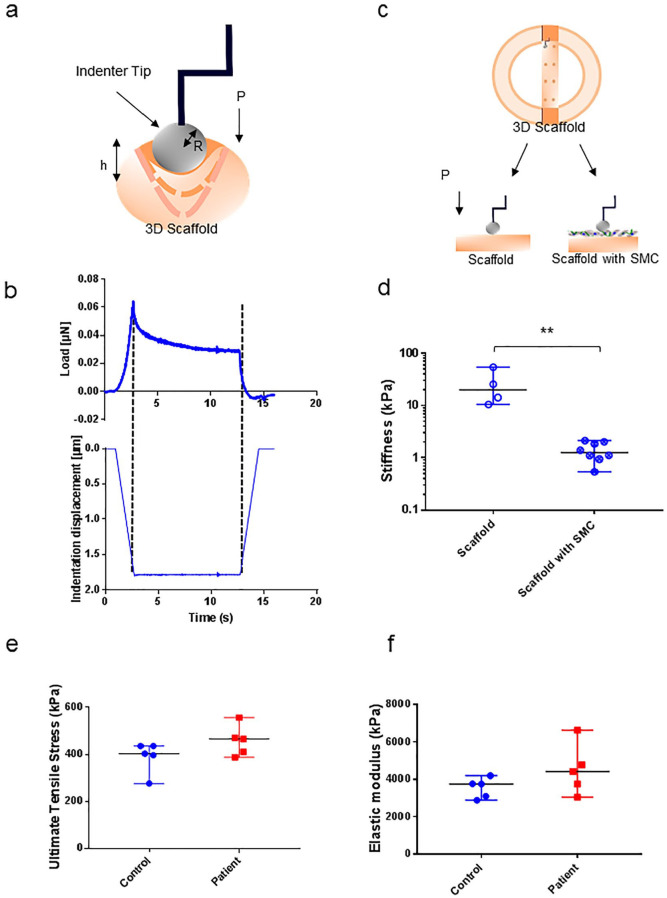
Mechanical properties and stiffness of the vascular 3-dimensional (3D) smooth muscle cell (SMC) culture. (a) Schematic representation of a spherical tip indenting the surface of the vascular scaffold with constant pressure. (b) Stress-relaxation curve of constant pressure indentation of a scaffold with cells. Upper graph represents the force loading curve (µN), lower graph represents the constant indentation depth applied to the sample (µm). (c) Schematic representation of scaffold stiffness assessment using nanoindentaton and recorded diverse response from scaffolds with and without SMC. (d) Differences in stiffness of scaffold with and without cells (Pa, p<0.004) measured by nanoindentation. Each data point represents 1 measurement, obtained by indenting the surface. (d) Differences in tensile stress between scaffolds seeded with control and abdominal aortic aneurysm (AAA) patient SMC, measured by uniaxial tensile test (kPa). (e, f) Differences in elastic modulus between scaffolds seeded with control and AAA patient SMC, measured by uniaxial tensile test (kPa). Each data point represents the mean of measurements performed in 3 scaffolds.

Micromechanical properties of scaffolds without cells and scaffolds seeded with SMC were measured using nanoindentation. Viscoelastic properties of the scaffold were assessed by applying longer constant pressure with a nanoindenter (Figure 3a). The mechanical load of the sample decreased in time under constant pressure, exhibiting viscoelastic behavior, characteristic for biological tissues^22^ (Figure 3b). Scaffold stiffness was assessed by indenting samples with and without SMC (Figure 3c). Median stiffness of the scaffold without cells (19.8, range 10.5–54.3 kPa) was almost 20-fold higher compared with the median stiffness of the scaffold seeded with SMC (1.3 kPa; range 0.5–2.1 kPa; p=0.004, Figure 3d).

### Mechanical Properties of the 3D SMC Cultures

Mechanical properties of the scaffolds were assessed after 5 weeks of culturing SMC on the 3D scaffold and ultimate tensile stress was compared between samples seeded with control and patient SMC. Similar tensile stress was observed in the patient group, with a median response of 460 kPa (380–550 kPa) as in the control group, median 400 kPa (range 280–440 kPa, Figure 3e). The elastic modulus was presented as the recorded median elasticity of the AAA patient group: 442 kPa (range 306–664 kPa) vs controls median 376 (range 289–420; Figure 3f).

### Control SMC Produce More Fibrillin-1 Networks Compared With AAA Patients

**Figure 4.**
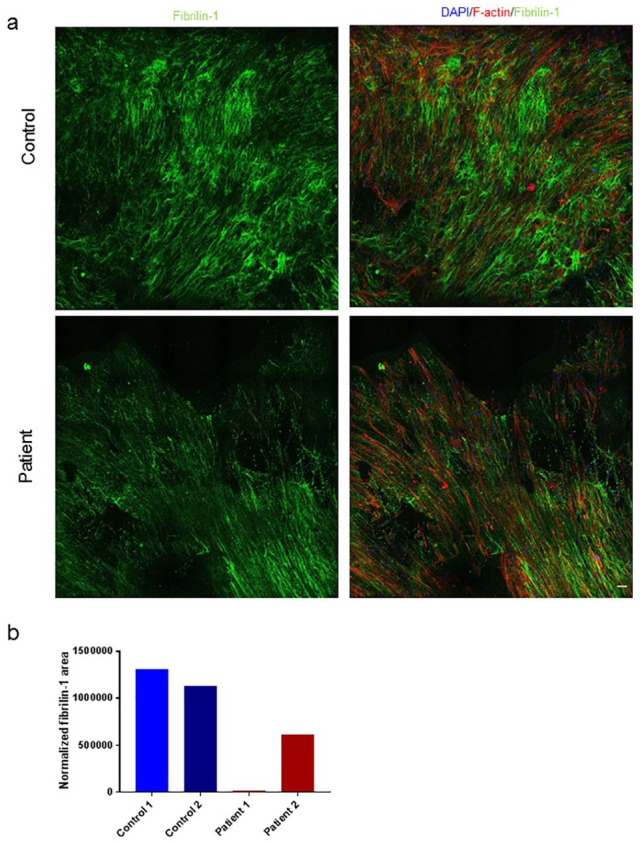
Fibrilin-1 organization differs in cultures with control and abdominal aortic aneurysm (AAA) patient smooth muscle cell (SMC). Samples were immunostained for F-actin (red), DAPI (blue) and Fibrilin-1 (green). (a) Representative images of Fibrilin-1 staining in controls and patients after 3 weeks of culturing on 3-dimensional (3D) scaffolds. (b) Fibrilin-1 production during after 5 weeks was measured on glass coverslips in control (n=2, blue) and patient (n=2, red) SMC. (d) Bar graph represents the mean per cell line. Scale bar: 50 µm.

In order to see if we could study ECM networks in our novel 3D model, we have quantified Fibrillin-1 in controls (n=2) and AAA patients (n=2) after 5 weeks of culturing on 3D scaffolds. A trend of higher Fibrillin-1 production is present in the control group (Figure 4a and b; representative 3D structures of control and patients SMC and ECM are shown in Video 1 and Video 2, respectively).

## Discussion

Defects in SMC and elastic fibers of the ECM, which consequently lead to altered cell-matrix interaction, play a role in thoracic aortic aneurysm formation. This underscores the relevance of explaining the dynamics between SMC and ECM in the context of aortic disease.^23^ However, identifying the mechanisms behind these defects is hindered by the lack of relevant bioengineered models. In the present study, we created a 3D vascular scaffold to study SMC and ECM organization in AAA. Our scaffolds demonstrate a multilayered SMC culture, aiming to achieve morphological and mechanical properties similar to those of biological vascular tissues and mimic interaction between the medial and intimal layer. To the best of our knowledge, this is the first bioengineered model with patient-specific SMC of healthy individuals and AAA patients for the study of AAA.

Current bioengineering innovations in 3D printing are not progressive enough to print highly complex and accurate models of the aneurysmal aortic wall.^24^ As our construct represents a novel model, we compared our findings regarding SMC behavior with known and well-established 2D cultures for validation. Culturing cells on a scaffold allows for a more robust construction, consisting of more successive layers of SMC, which closely resembles the aorta, and is not threatened by cell detachment like 2D cultures. Therefore, we opted to use PLGA, a biodegradable material, which was previously known as suitable material for vascular scaffolding using SMC.^25^ To the best of our knowledge, this is the first model that is bioengineered with patient specific SMC of both healthy individuals and AAA patients, and thus provides a unique possibility to study patient-specific physiological processes in AAA involving SMC.

As opposed to the 2D cultures where we observed a monolayer of EC, the co-culture on the 3D scaffolds appeared to be less uniform. The EC possibly filled the gaps in between the SMC layers, forming this distinct morphology. Similar structures were observed in the presence of fibroblast-conditioned medium, indicating that the tube-like cell could also be a product of the signaling molecules excreted by the larger number of SMC on the scaffold.^26^ Another explanation could be that the multilayered SMC provide a substrate of different stiffness than the few layers of SMC on the stiff glass coverslip; similar tube-like structures were observed in lower percentage collagen gels.^27^

Aortic stiffening occurs primarily in regions of the aorta that are prone to aneurysmal dilation^28^. This supports our findings that scaffolds seeded with SMC derived from AAA patients demonstrate a trend of increased stiffness compared with controls. The stiffness measurements revealed stiffness ranges common for biological ex vivo vessels and viscoelastic properties typical for living tissue.^22,29^ Moreno-Flores et al^30^ measured stress-relaxation as parameters of cellular viscoelasticity, demonstrating the same 3-fold decrease in force (Figure 3b). Fibrillin-1 is a found in the elastic fiber microfibrils in the extracellular matrix of the aorta.^31^ Our results show a trend of decrease in fibrillin-1 in AAA patients compared with controls, in accordance with known ECM remodeling in AAA. To draw definitive conclusions on the mechanical properties of scaffolds seeded with patient SMC and statistically significant data, larger groups are needed. We will expand the group sizes for these measurements in our follow-up studies.

A shortcoming of this study is the small group size and low number of SMC donors used per experiment. However, as the primary goal was to share our design of 3D vascular scaffolds and hint the prospective applications for discriminating between controls and AAA patients, we present the findings within the current group sizes. At its current state, the method requires a few months to study the patient cells, since a lot of cells are required per scaffold and it takes time to proliferate them. An additional word of caution: As the biopsies were obtained during open aneurysm repair, we did not have control over the aortic site from which the biopsy derived due to prioritizing patient safety. Yet we can confidently state that all the patient biopsies are derived from the abdominal region of the aorta belonging to the dilated and diseased region of the aneurysmal sac, in most cases from the ventral side.

Upon validating our 3D scaffolds, we aim to use our novel method to investigate various AAA related risk factors, such as male gender, smoking and diabetes. We plan to set up follow-up studies in which we will compare scaffolds seeded with control cells and, for example, patients who currently smoke and currently do not smoke, and investigate differences using the presented assays. Furthermore, we plan to increase the complexity of our model by generating a tubular scaffold using a bioreactor, to add additional parameters such as flow, and increase the resemblance of our model to a vessel.

## Conclusion

We demonstrate that SMC can form complex cultures in vitro consisting of patient-specific cells and the ECM they produced. We measured mechanical and biological properties comparable to the aortic wall and we showed the possibility to co-culture SMC and EC in our setup. Although future investigation is needed to perfect the model design and increase its size and complexity, we deem that it represents a valuable preclinical model of AAA with applications in both translational research and therapy developments.

## Supplemental Material

sj-pdf-1-jet-10.1177_15266028211009272 – Supplemental material for Patient-Specific 3-Dimensional Model of Smooth Muscle Cell and Extracellular Matrix Dysfunction for the Study of Aortic Aneurysms Click here for additional data file. Supplemental material, sj-pdf-1-jet-10.1177_15266028211009272 for Patient-Specific 3-Dimensional Model of Smooth Muscle Cell and Extracellular Matrix Dysfunction for the Study of Aortic Aneurysms by Natalija Bogunovic, Jorn P. Meekel, Jisca Majolée, Marije Hekhuis, Jakob Pyszkowski, Stefan Jockenhövel, Magnus Kruse, Elise Riesebos, Dimitra Micha, Jan D. Blankensteijn, Peter L. Hordijk, Samaneh Ghazanfari and Kak K. Yeung in Journal of Endovascular Therapy

sj-pdf-2-jet-10.1177_15266028211009272 – Supplemental material for Patient-Specific 3-Dimensional Model of Smooth Muscle Cell and Extracellular Matrix Dysfunction for the Study of Aortic Aneurysms Click here for additional data file. Supplemental material, sj-pdf-2-jet-10.1177_15266028211009272 for Patient-Specific 3-Dimensional Model of Smooth Muscle Cell and Extracellular Matrix Dysfunction for the Study of Aortic Aneurysms by Natalija Bogunovic, Jorn P. Meekel, Jisca Majolée, Marije Hekhuis, Jakob Pyszkowski, Stefan Jockenhövel, Magnus Kruse, Elise Riesebos, Dimitra Micha, Jan D. Blankensteijn, Peter L. Hordijk, Samaneh Ghazanfari and Kak K. Yeung in Journal of Endovascular Therapy

sj-pdf-3-jet-10.1177_15266028211009272 – Supplemental material for Patient-Specific 3-Dimensional Model of Smooth Muscle Cell and Extracellular Matrix Dysfunction for the Study of Aortic Aneurysms Click here for additional data file. Supplemental material, sj-pdf-3-jet-10.1177_15266028211009272 for Patient-Specific 3-Dimensional Model of Smooth Muscle Cell and Extracellular Matrix Dysfunction for the Study of Aortic Aneurysms by Natalija Bogunovic, Jorn P. Meekel, Jisca Majolée, Marije Hekhuis, Jakob Pyszkowski, Stefan Jockenhövel, Magnus Kruse, Elise Riesebos, Dimitra Micha, Jan D. Blankensteijn, Peter L. Hordijk, Samaneh Ghazanfari and Kak K. Yeung in Journal of Endovascular Therapy
