# Supplementary material for: Patient-Specific 3-Dimensional Model of Smooth Muscle Cell and Extracellular Matrix Dysfunction for the Study of Aortic Aneurysms
Source: J Endovasc Ther. 2021 Apr 26;28(4):604–13. doi: 10.1177/15266028211009272 (PMC8276336; doi:10.1177/15266028211009272)
Supplement: sj-pdf-1-jet-10.1177_15266028211009272 – Supplemental material for Patient-Specific 3-Dimensional Model of Smooth Muscle Cell and Extracellular Matrix Dysfunction for the Study of Aortic Aneurysms [file sj-pdf-1-jet-10.1177_15266028211009272.pdf]

“Supplementary Figure 1. PLGA scaffold seeding.

a)SEM images of 3D electrospun, patterned poly-lactide-co-glycolide scaffold (left panel: 500x magnification; right panel: 2000x magnification; scale bar: 10µm). b) Schematic representation of centralized SMC seeding area on the 3D scaffold (19 x 5 mm).”
