# Supplementary figures and images for: Patient-Specific 3-Dimensional Model of Smooth Muscle Cell and Extracellular Matrix Dysfunction for the Study of Aortic Aneurysms
Source: J Endovasc Ther. 2021 Apr 26;28(4):604–13. doi: 10.1177/15266028211009272 (PMC8276336; doi:10.1177/15266028211009272)

a

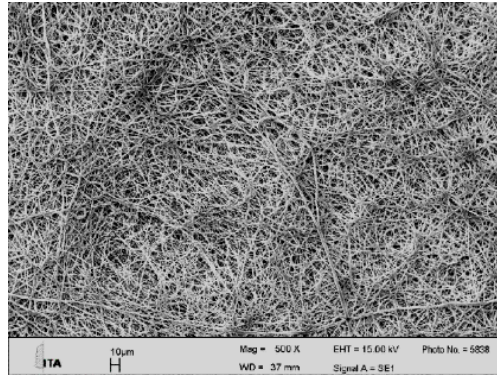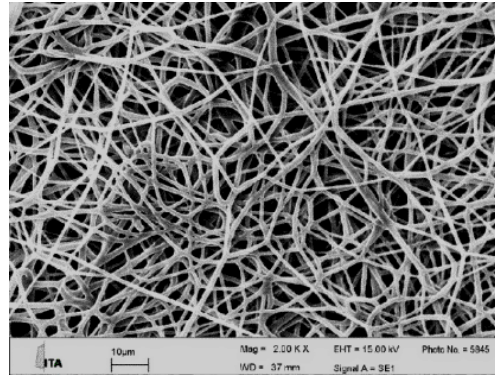

b

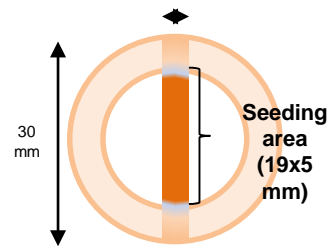

Supplement: sj-pdf-2-jet-10.1177_15266028211009272 – Supplemental material for Patient-Specific 3-Dimensional Model of Smooth Muscle Cell and Extracellular Matrix Dysfunction for the Study of Aortic Aneurysms [file sj-pdf-2-jet-10.1177_15266028211009272.pdf]
