# Supplementary material for: Patient-Specific 3-Dimensional Model of Smooth Muscle Cell and Extracellular Matrix Dysfunction for the Study of Aortic Aneurysms
Source: J Endovasc Ther. 2021 Apr 26;28(4):604–13. doi: 10.1177/15266028211009272 (PMC8276336; doi:10.1177/15266028211009272)
Supplement: sj-pdf-3-jet-10.1177_15266028211009272 – Supplemental material for Patient-Specific 3-Dimensional Model of Smooth Muscle Cell and Extracellular Matrix Dysfunction for the Study of Aortic Aneurysms [file sj-pdf-3-jet-10.1177_15266028211009272.pdf]

**Cell line**	**Age (years)**	**Gender**	**Aneurysm diameter (cm)**	**Rupture**
Control 1	69	M	N/A	N/A
Control 2	59	M	N/A	N/A
Control 3	44	F	N/A	N/A
Control 4	50	M	N/A	N/A
Control 5	68	M	N/A	N/A
Control 6	59	M	N/A	N/A
Control 7	50	M	N/A	N/A
AAA patient 1	68	M	55	No
AAA patient 2	64	M	63	No
AAA patient 3	75	F	70	Yes
AAA patient 4	63	F	63	No
AAA patient 5	74	F	55	No
AAA patient 6	74	M	100	Yes
AAA patient 7	60	M	56	No
AAA patient 8	65	M	94	No

Supplementary Table 1. **Clinical characteristics of controls and AAA patients.** Age and gender are shown for all controls and patients. Aneurysm diameter and indication of rupture are shown for the patients.
